# Creep Testing of Thermoplastic Fiber-Reinforced Polymer Composite Tubular Coupons

**DOI:** 10.3390/ma13204637

**Published:** 2020-10-17

**Authors:** Hai Giang Minh Doan, Pierre Mertiny

**Affiliations:** Department of Mechanical Engineering, University of Alberta, 9211-116 St., Edmonton, AB T6G 1H9, Canada; hdoan@ualberta.ca

**Keywords:** thermoplastic fiber-reinforced polymers composites, time-dependent behavior, creep testing

## Abstract

Thermoplastic fiber-reinforced polymer composites (TP-FRPC) are gaining popularity in industry owing to characteristics such as fast part fabrication, ductile material properties and high resistance to environmental degradation. However, TP-FRPC are prone to time-dependent deformation effects like creep under sustained loading, which can lead to significant dimensional changes and affect the safe operation of structures. Previous research in this context has focused, mainly, on testing of flat coupons. In this study, a creep testing method for TP-FRPC tubular coupons was developed. Specimens were fabricated using tape winding and subjected to well-defined loading conditions, i.e., pure hoop tensile and pure axial compressive stress. Strain gauges and digital image correlation were both employed for strain measurements and were found to be in good agreement. The evolution of strain rate, Poisson’s ratio and creep compliance were investigated. The prediction of experimental data by the Burgers model and the Findley’s power law model were explored. The research findings suggest that the developed experimental and analysis approach provides valuable information for the design of material systems and structures.

## 1. Introduction

Fiber-reinforced polymer composite (FRPC) structures are increasingly being adopted by industry, including FRPC pressure piping [1]. Compared to comparable steel structures, FRPC pressure components can provide improvements in terms of safety, cost and environmental performance. For example, fluids transported in pipes can be corrosive [2], and failure of corroded pipes can have major safety and environmental impacts. Pipeline corrosion had an estimated annual cost of USD 7 billion in the USA alone (based on a 2001 NACE study) [3]. Corrosion concerns can be alleviated using FRPC materials which have superior corrosion resistance compared to steel [1]. FRPC pipes also possess a higher strength-to-weight ratio and greater flexibility; these characteristics help reduce installation and transportation costs [4]. The combination of weight reduction and corrosion resistance have made FRPC piping especially advantageous in deep-sea oil and gas applications [5]. Subsea oil fields are being developed at depths up to 3000 m [6]; the use of FRPC reduces the weight of both the pipe and supporting structures resulting in substantial cost savings [5]. Not limited to the oil and gas industry, FRPC pipes are also used in water distribution [7], sewage [8], and geothermal heating systems [9]. FRPC piping has traditionally been based on thermoset polymers, with thermoplastic (TP) matrix materials emerging as an alternative in recent years, owing to even lower material costs for some material systems such as glass fiber reinforced high density polyethylene (GFR-HDPE), fast composite fabrication, and ductile material properties.

However, FRPC structures, and especially those having a TP matrix, are susceptible to time-dependent deformation effects, such as polymer creep and fiber realignment. Creep is the continuous deformation of a material subjected to sustained loading [10], while fiber realignment is the reorientation of a fiber reinforcement phase under the applied load [11]. Both phenomena can affect the safety and reliability of the structure over time. Understanding the long-term behavior of the material under sustained loading is a necessary part of designing FRPC structures for long-term operation. The expected lifetime of certain FRPC piping systems is 20 years [12]; therefore, they may experience significant deformation due to creep during this time.

**Figure 1:**
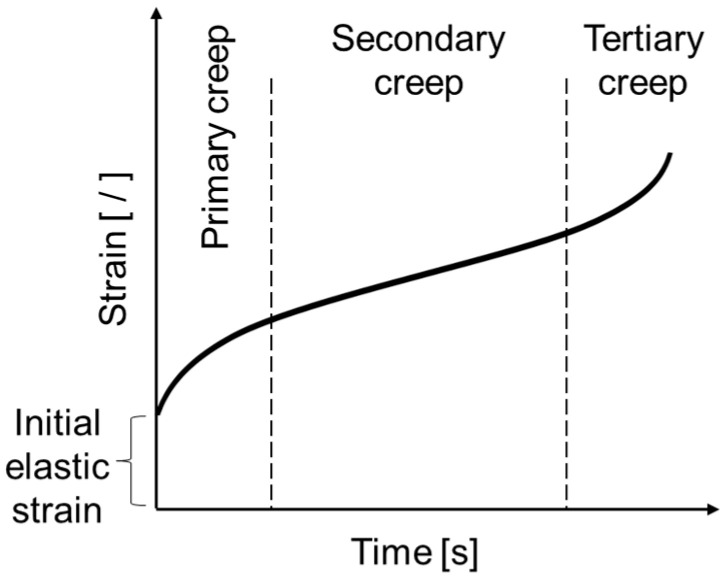
Three stages of creep (reproduced from [16]).

There are three phases of creep in polymeric materials, i.e., primary, secondary and tertiary creep [13], as indicated in Figure 1. Upon applying load, an initial elastic strain is caused by the changing bond distances and angles of the polymer chains [14]. The initial strain is followed by the primary and secondary—also called steady-state—creep phases. The continued application of stress causes the polymer chains to untangle and rearrange resulting in greater alignment of the chains [13,14]. The strain rate increases during tertiary creep and can eventually lead to failure [15].

**Figure 2:**
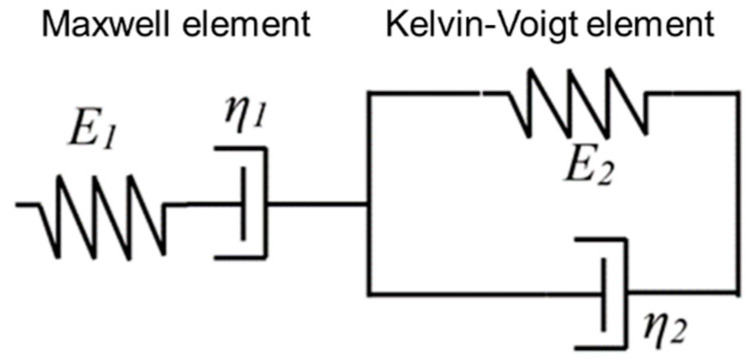
Representation of the Burgers model using Maxwell and Kelvin-Voigt elements.

Long-term creep tests lasting up to 10,000 h [17,18] have been conducted to quantify the effect of creep on failure strength and lifetime of pipe structures. However, long-term creep testing is resource intensive and, therefore, models have been developed, based on short-term creep experiments, to predict the long-term creep behavior of the material [19]. The Burgers model (BM) and Findley’s power law model (FPLM) have been proposed to predict material viscoelastic behavior [13,20,21,22]. The BM, which is only valid for linear viscoelastic behavior, combines the Maxwell and Kelvin elements in series [23] as shown in Figure 2. It does not consider inertial effects. The strain-time relationship under a constant stress for the BM is shown in Equation (1). The FPLM, expressed in Equation (2), can represent nonlinear viscoelastic behavior. It was empirically derived and found to model creep in unreinforced plastics but several researchers have applied it to polymer composites as well with good agreement [23,24].

(1) $$\varepsilon \left(t\right)=\;\frac{\sigma_{0}}{E_{1}}+\frac{\sigma_{0}}{\eta_{1}}t+\frac{\sigma_{0}}{E_{2}}\left(1-e^{-\frac{E_{2}t}{\eta_{2}}}\right)$$

where ε(t) is the strain at given time *t*; *E*_1_, *E*_2_, *η*_1_ and *η*_2_ are material-dependent parameters (i.e., the Maxwell and Kelvin-Voigt spring moduli, and the Maxwell and Kelvin-Voigt dashpot viscosities, respectively), which can be found by curve fitting creep test data.

(2) $$\varepsilon \left(t\right)=\;\varepsilon_{0}+\varepsilon^{+}t^{n}$$

where $\varepsilon_{0}$ is the initial elastic strain; $\varepsilon^{+}$ is a function of temperature, material and load; and n is a material dependent parameter.

Researchers have used experimental and modelling approaches to study creep in engineering materials. However, there is comparatively limited research on thermoplastic FRPC (TP-FRPC) since it is a less mature technology. TP-FRPC provide better ductility and impact performance, a long polymer phase storage life, lower material costs for some material systems, fast part fabrication, and more available repair options compared to their thermoset counterparts [25]. A review of the technical literature on TP-FRPC research revealed several studies for testing of flat coupons [26,27] which can result in stress concentrations at the edges, known as free-edge effects [28]. It is questionable for such testing to be representative of the stresses experienced in the bulk material. There are standards for long-term material testing of FRPC, e.g., ISO 899 [29] relates to flexural creep in dumbbell-shaped specimens, ISO 7509 [30] is concerned with the long-term time-to-failure of thermoset FRPC pipe subjected to internal pressure, and ISO 7684 [31] deals with the creep of pipes subjected to an external compressive force. Yet, no standards were found that directly relate to creep in TP-FRPC piping and tubular components under biaxial stress and internal pressure loading.

The work in the present study is part of a larger research project in which university and industry partners collaborate to address the issue of time-dependent deformation in TP-FRPC piping structures. To aid in continuous improvement efforts and design of new products, modelling techniques are being developed that are capable of considering the combined effects of polymer creep and fiber realignment. Essential for these efforts are experimental data to calibrate and validate the models. To achieve this objective, tubular coupons made from GFR-HDPE were manufactured using a custom tape winding setup. Using tubular coupons eliminates undesirable free-edge effects, yet, fabrication and testing of such samples is technologically involved. Microstructure analysis, bond uniformity tests and dimensional measurements were used to verify specimen quality. Using specialized testing equipment, the tubular specimens were subjected to creep testing under pure hoop tensile stress and then pure axial compressive loading conditions to investigate their time-dependent performance. Two independent measurement techniques, strain gauges and digital image correlation (DIC), were used to verify the accuracy of strain measurements. Changes in strain rate, Poisson’s ratio and creep compliance were investigated. The BM and FPLM were used to fit the experimental data. Test and model predictions were contrasted to identify suitable techniques for the engineering design of TP-FRPC pressure piping.

## 2. Materials and Methods

### 2.1. Fabrication of TP-FRPC Tubular Coupons

**Figure 3:**
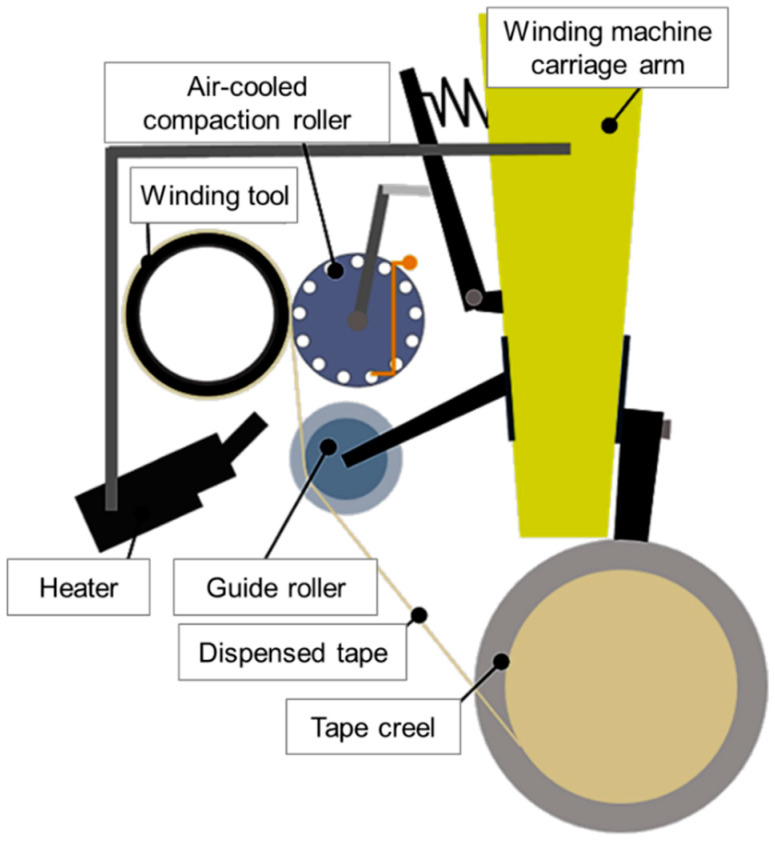
Schematic of tape winding setup.

Unidirectional GFR-HDPE tape (Taizhou Jiadebao Technology Co., Ltd., Taizhou, China) with thickness of 0.33 mm and width of 49 mm was used to fabricate the tubular coupons. The fiber-to-matrix weight fraction of the tape was verified by resin burnout tests to be within manufacturer specifications of 60 ± 2%, with a composite material density 1.56 g/cm^3^. A filament winding machine (WMS-4 Axis, McClean-Anderson, Schofield, WI, USA) was modified to enable fabrication by tape winding. Specifically, additional equipment for holding a tape creel, guiding and heating the tape, and consolidating tape on the fabricated part was added to the existing machine. The prototyping setup is schematically depicted in Figure 3. From the tape creel, the tape is directed by a guide roller. Tape tension was applied mechanically via a friction brake. A hot-air blower (Hotwind Premium, Leister Technologies AG, Kaegiswil, Switzerland) was used to heat the tape as it approaches an air-cooled aluminum compaction roller. The compaction roller applies pressure to the tape as it is wound around the rotating winding tool to build up the composite tubular part layer by layer. The compaction force applied by the compaction roller is spring-controlled. For the present study, the winding angle for tubular coupons was chosen to be −45°/+45°. Considering both the tube hoop and axial directions, this winding angle produces matrix-dominant properties [27] and the lowest Young’s modulus as compared to other angle-ply configuration [32]. As such, it was expected that, for this winding angle, the material creep response, which is highly dependent on the matrix properties, would be most pronounced when applying pure hoop and pure axial loading conditions during testing.

Tubular coupons were fabricated on an HDPE liner tube covering an aluminum winding tool. The use of a low thermal conductivity liner reduced the amount of heat conducted away from the tape as it was placed on the winding tool. As it was not desired for the tape to bond to the liner, aluminum foil coated with a release agent (MAC-860, McLube, Aston, PA, USA) covered the liner tube. The winding speed for the initial tape layer was significantly higher than for subsequent layers, with heating just sufficient to allow the tape to conform to the tubular shape without any buckling. Given the 49 mm tape width, three passes were required to complete one layer. For subsequent layers, the winding speed was reduced to allow the tape surface to reach its melting temperature of approximately 130 °C prior to tape placement. The tape was visually monitored during winding to check for adequate melting. Note that a thermal imaging camera was employed in initial winding trials (FLIR E60, FLIR Systems, Wilsonville, OR, USA), providing accurate tape temperature measurements, in order to adjust appropriate heater and winding machine settings. The tape deposition process continued until 10 tape layers were placed. Two batches of tubular coupons were produced as per the processing parameters listed in Table 1, i.e., heating was raised with sequent sets of deposited layers. Upon reaching the desired tube thickness, additional passes of the compaction roller were completed applying heat and pressure to promote part compaction and create a smooth tube surface. 

After tape winding, the winding tool was removed, and tubular coupons were cut from the fabricated part to a length of 203.2 mm (8”). After cutting, the HDPE liner tube and aluminum foil could be removed easily. Short ring sections with length of 12.7 mm (0.5”) were also cut adjacent to each tubular coupon. The ring samples were used to verify inter-layer bond quality and tape consolidation, which was accomplished by crushing the rings in a vise and verifying the absence of composite delamination.

The average inner diameter of tubular coupons was 61.81 and 61.88 mm with a standard deviation of 0.06 and 0.05 mm for Batch #1 and #2, respectively; the average wall thickness was correspondingly 3.65 and 3.61 mm, both with a standard deviation of 0.03 mm. Based on a two-mean, unequal variance t-test, the difference between average inner diameter and wall thickness between Batch #1 and #2 was statistically insignificant for a 95% confidence interval. It is interesting to note that the thickness of the 10-layer tubes (approximately 3.6 mm) was greater than the cumulative thickness for 10 layers of unprocessed tape (3.3 mm), which is likely caused by a variation in tape thickness introduced during tensioning and heating as well as lateral tape compaction during the winding process. The tape was placed in a “lag” winding mode, meaning the tape from subsequent passes was placed against previously laid tape. Increased thickness of final product dimensions caused by the manufacturing process has been observed previously in other tape placement applications [33].

**Figure 4:**
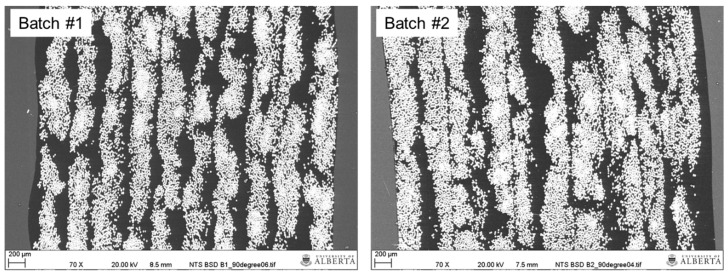
Representative microstructures of fabricated tubular coupons.

Scanning electron microscopy was used to inspect the microstructure of the final product using an EVO LS15 EP-SEM instrument (Zeiss, Oberkochen, Germany) operated in backscatter mode at a voltage of 20 kV. Composite samples were cast in cold-cure epoxy and polished to create microscopy specimens. The latter were carbon-coated (EM SCD005, Leica Microsystems, Wetzlar, Germany). Figure 4 shows representative microstructures for Batch #1 and #2, where the fibers, matrix and encasing epoxy appear in as white, dark grey and light grey areas, respectively. The analyses of microscope images revealed negligible void content in the final product. The 10 tape layers are recognizable in Figure 4, with matrix-dominant and fiber-dominant regions interspersed throughout the structure. In certain areas, the tape layers blend together resembling the microstructure observed by Mazumdar and Hoa [34]. Blending of layers may be the result of the applied processing temperature and compaction pressure, causing viscous or melted HDPE to be displaced as tape is being placed. This supposition is supported by the observation that fiber layers are more closely compacted for the Batch #2 sample where higher processing temperatures were applied compared to the Batch #1 sample.

### 2.2. Preparation of Tubular Coupons

To mount a tubular coupon into the testing apparatus, custom steel end fittings were adhesively bonded to the coupon extremities using a two-part adhesive (DP460, 3 M, Maplewood, MN, USA). Mating surfaces were cleaned with abrasive pads and acetone, followed by a flame treatment (propane gas) to activate the polymer and promote bonding before applying adhesive and insertion into the end fitting assembly. A fillet was allowed to form to reduce the stress concentration at the transition from end fitting to coupon.

**Figure 5:**
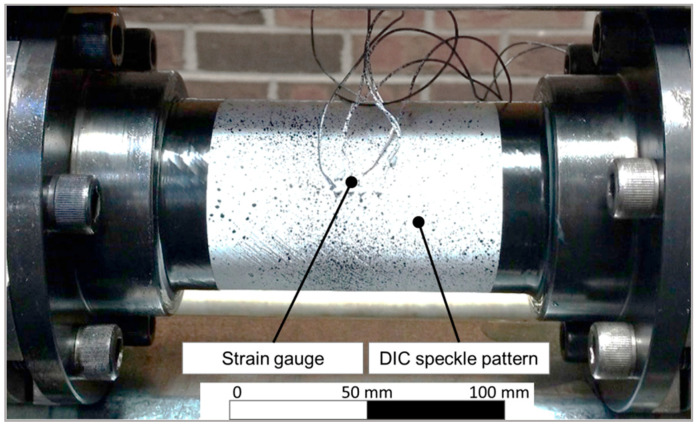
Tubular coupon mounted into testing apparatus prior to testing.

A tee-rosette strain gauge (CEA-06-250UT-350, Micro-Measurements, Raleigh, NC, USA) was attached to the middle of the coupon and aligned to simultaneously measure hoop and axial strains. A speckle pattern, required for the DIC strain measurement, was applied using white and black spray paint. Figure 5 shows a representative specimen prior to creep testing.

### 2.3. Testing Procedure and Data Acquisition

A multi-axial testing apparatus connected to an analogue micro-controller (Model 458.10 MicroConsole, MTS Systems, Eden Prairie, MN, USA) was used to apply the test loading conditions. The servo-hydraulic testing apparatus can apply axial force and internal pressure in a controlled manner. Hydraulic oil is the medium for pressurization. All tests were conducted at room temperature. The first test condition for the tubular specimens was applying pure hoop tensile stress. For this purpose the specimen is deemed a closed-ended pressure vessel, and hence, axial forces are induced in the specimen during pressurization. A pure hoop loading condition was achieved by applying an internal pressure, $P_{i}$, while the axial actuator applied a compressive axial force, $F_{A}$, to compensate for the pressure-induced axial force. The hoop and axial stresses, as well the magnitude of the axial force that was applied by the actuator, were determined based on Equations (3) and (4).

(3) $$\sigma_{\mathrm{hoop}}=\frac{P_{i}d_{i}-P_{o}d_{o}}{d_{o}-d_{i}}$$

(4) $$\sigma_{\mathrm{axial}}=\frac{\left(P_{i}\pi d_{i}^{2}-P_{o}\pi d_{o}^{2}\right)}{\pi (d_{o}^{2}-d_{i}^{2})}-F_{A}\frac{4}{\pi \left(d_{o}^{2}-d_{i}^{2}\right)}$$

where $\sigma_{\mathrm{hoop}}$ and $\sigma_{\mathrm{axial}}$ are the hoop and axial stress; $d_{i}$ and $d_{o}$ are the tube inner and outer diameter, respectively. The gage pressure of the testing equipment was used for $P_{i}$; therefore, the outer pressure, $P_{o}$, was considered to be zero.

In this study, the purpose for the experiments is to provide creep data as input to calibrate and verify numerical models. Therefore, well-defined loads that induce creep in the material need to be imposed upon the tubular coupons. HDPE has been demonstrated to creep at stresses as low as 2 MPa at room temperature [35]. It was expected that GFR-HDPE would experience creep at comparative low stress levels as well, assuming the loading direction deviates sufficiently from the directions of the fiber reinforcement, since creep behavior is predominantly determined by the properties of the matrix material. Nevertheless, stress values suitable for pure HDPE were deemed too low, given that the polymer phase was reinforced with continuous fibers at a considerable volume fraction (i.e., 60%). Also, stress values below a certain level pose challenges in terms of the operating range of the testing equipment. Therefore, a stress value of 10 MPa was chosen.

Each creep tests had a 10-s ramp-up period. Three samples from Batch #2 (H001, H002 and (H003) and two specimens from Batch #1 (H004 and H005) were tested. (Note that for clarity, not all test data are plotted as part of subsequent analyses.) Tests with different durations were conducted: short-term (approximately 2 h), intermediate (4 h) and longer-term (7 h). Virgin specimens were used for the pure hoop tests. Specimens were then re-used for pure axial compression testing after allowing the specimen to rest in the testing apparatus overnight. A total of three pure axial compression tests were conducted, i.e., one specimen each from short-term, intermediate and longer-term pure hoop testing. For the pure axial compression tests, specimens were not pressurized; only a compressive axial load, equivalent to 5 MPa stress, was applied. Table 2 shows the test matrix for the creep experiments. Tests with the suffix “b” denote pure axial compression tests. Test durations for the pure axial tests were either 4 h or 6 h.

As mentioned above, strain gauges and DIC were used to measure hoop and axial strains experienced by the specimens, with a sampling frequency of 1 and 0.1 Hz, respectively. The specimen strain gauge was connected in series to a dummy gauge and signal conditioner to form a Wheatstone quarter-bridge arrangement with temperature compensation. The dummy gauge was the same type as the specimen gauge and applied to a strip of unprocessed GFR-HDPE tape; the gauge was further orientated 45° to the fibers so it would have the same orientation with respect to the fibers as the one on the test specimen.

The components of the DIC system consisted of two camera lenses (28–85 mm, Nikon, Tokyo, Japan) each attached to CCD cameras (Pike F-421, Allied Vision Technologies GmbH, Stadtroda, Germany) and two LED lights. The distance between the cameras and specimen was approximately 0.8 m. The focal length and aperture of the lenses were set to 50 mm and f3.5, respectively. The camera components were placed on a tripod. For safety reasons, the tests were conducted with a polycarbonate shield surrounding the specimen. The placement of the shield between the cameras and specimen resulted in higher calibration error but was still within acceptable limits as stipulated by the DIC system manufacturer.

**Figure 6:**
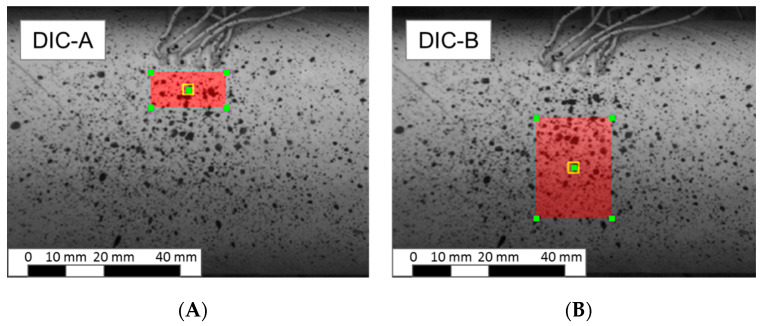
Sample photographs of speckle pattern used for digital image correlation (DIC) strain measurements: DIC-A coinciding with strain gauge location (**A**), and large strain measurement area for DIC-B (**B**).

DIC data acquisition and post-processing was performed using the VIC-3D system (Correlated Solutions, Inc., Irmo, SC, USA). The use of the DIC technique allowed strain measurements in multiple areas of varying sizes on the specimen surface as opposed to the comparatively small area covered by the strain gauge. Measurement from the limited strain gauge area can be affected by irregularities in the tube or the tube surface, whereas DIC allows for a much larger measurement area and is thus less affected by local irregularities. For this study, two areas of interest were selected for DIC analysis. An area covering the strain gauge area was selected to allow a direct comparison with strain gauge measurements as shown in Figure 6A. This area viewed within the analysis software was 150 pixels wide and 70 pixels tall. Measurements from this area are referred to as DIC-A for the remainder of this paper. The second area, called DIC-B and shown in Figure 6B, was selected to be 10 pixels below the DIC-A area and was 150 pixels wide and 210 pixels tall. Note all strain measurements were made in the middle of the specimen to minimize any end effects.

In addition to strain measurements, initial elastic strain predictions were made based on micro-mechanical analysis and classical lamination theory [36], and predictions were compared with strain measurements. The elastic properties for the fiber reinforcement and HDPE matrix phase were not available from the tape manufacturer and, hence, the technical literature was perused to set reasonable ranges of maximum and minimum properties (as listed in Table 3) for the predictions. 

The longer-term effect of the applied loads on strain rate, Poisson’s ratio, and creep compliance for the tubular coupons was investigated. Strain rate, ${\dot{\varepsilon }}_{i}$, at a certain time, $t_{i}$, was calculate using a symmetric difference quotient approach as per Equation (5).

(5) $$\dot{\varepsilon_{i}}=\frac{\varepsilon_{i+100}-\varepsilon_{i-100}}{t_{i+100}-t_{i-100}}$$

where $\varepsilon_{i+100}$ and $\varepsilon_{i-100}$, and $t_{i+100}$ and $t_{i-100}$ are the strain and the time values at 100 s after and 100 s before time $t_{i}$, respectively.

Poisson’s ratio data was determined according to Equation (6). The initial Poisson’s ratio was predicted based the classical lamination theory [36] as shown in Equation (7). The derivation of this equation assumes a symmetric laminate, i.e., the coupling stiffness matrix [B] is zero. However, even though the present laminate is not symmetric, bending moments are considered negligible given the axisymmetric structure configuration (tube).

Finally, creep compliance, D(t), is the relationship between the strain, ε(t), and the constant stress, $\sigma_{0}$, experienced by the material as shown in Equation (8).

(6) $$\nu =-\frac{\varepsilon_{\mathrm{trans}}}{\varepsilon_{\mathrm{long}}}$$

(7) $$\nu_{xy}=-\frac{A_{12}^{*}}{A_{11}^{*}}$$

(8) $$D\left(t\right)=\frac{\varepsilon \left(t\right)}{\sigma_{0}}$$

where $\varepsilon_{\mathrm{trans}}$ and $\varepsilon_{\mathrm{long}}$ are correspondingly the strain perpendicular and in-line with the applied loading direction; and $A_{11}^{*}$ and $A_{12}^{*}$ are terms from the inverse of the extensional stiffness matrix [A] used in classical lamination theory.

## 3. Results and Discussion

### 3.1. Creep Strain Results

**Figure 7:**
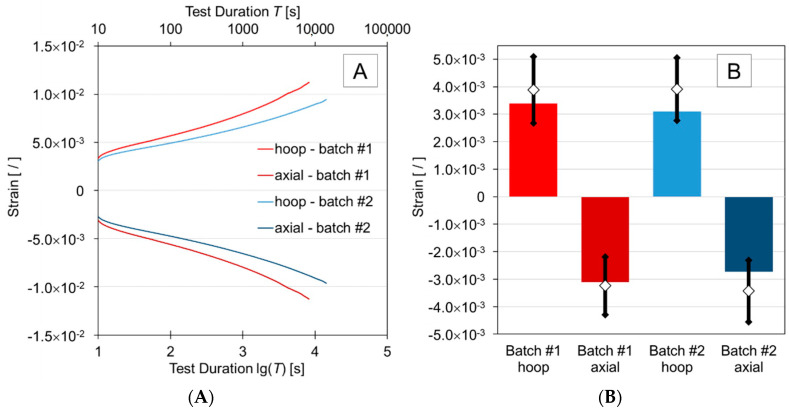
Strain from pure hoop tensile tests for representative samples from Batch #1 (H004) and Batch #2 (H001): (**A**) creep strain measured by strain gauges; (**B**) initial strain from strain gauges (solid bars) and predicted values based on data in Table 3 with maximums and minimums (error bars) and averages (diamond symbols).

Results obtained from strain gauge measurements for the 10 MPa pure hoop stress creep tests are depicted in Figure 7A. For clarity, only data for one specimen per batch is shown. In general, specimens within the same batch experienced similar levels of strain during these tests. Note that final strain values differed for the various tests given that test durations were not identical. Therefore, a reference time of 100 min was used to compare recorded creep strains at that test stage. The axial strain and hoop strain measured at 100 min for specimens H001, H002 and H003 were within 7% and 10% of each other, respectively. The measured axial and hoop strain for H004 and H005 were both within 7% of each other. It was concluded that repeatable creep strain values were observed for each batch. Moreover, despite the approximations made for the prediction of initial strains, and using methods assuming ideal conditions (micro-mechanical analysis and classical lamination theory), one can observe in Figure 7B that measured and predicted values are in reasonable agreement.

As shown in Reference [35], various grades of HDPE (but not necessarily a material identical to the composite matrix used in the present study) subjected to 10 MPa tensile stress, at room temperature, experienced over 0.02 mm/mm strain at the 1-h mark of creep testing. In comparison, hoop strains measured in the present study are in the order of 0.008 mm/mm after the same time period. This difference demonstrates the significant improvement in creep resistance that is imparted by the reinforced polymer as compared to the pure polymer.

As depicted in Figure 7A, specimens from Batch #1 (H004 and H005) experienced higher levels of initial elastic strain and final creep strain than those from Batch #2 (H001, H002, and H003). While dimensions, fiber volume fraction and void content of specimens in the two batches are practically identical, the differences in strain may be explained by differences in crystallinity. Agarwal et al. [37], as well as Mazumdar and Hoa [34], attributed changes in crystallinity of the polymer matrix to the annealing experienced by the tape as it is heated and reheated during the tape winding process. Agarwal et al. observed higher heat input during winding of carbon fiber reinforced polyether ether ketone tape resulted in a higher percentage of crystallinity in the finished product. Polyether ether ketone and HDPE are both semi-crystalline thermoplastic polymers. Additionally, it has been demonstrated that different cooling rates of melted HDPE resulted in different crystalline structures [38]. In this study, the GFR-HDPE tape received different heat inputs during the winding of each batch and was allowed to cool to room temperature. It is plausible to conclude that this process resulted in different degrees of crystallization in the finished product. A greater level of crystallinity corresponds to a greater number of secondary valence bonds between molecules [14], which explains the greater resistance to creep [27]. While differential scanning calorimetry (DSC) tests were not conducted to verify the crystallinity of the specimens, since this was not considered to be within scope of the project, DSC analysis could be conducted on specimens as part of future work.

**Figure 8:**
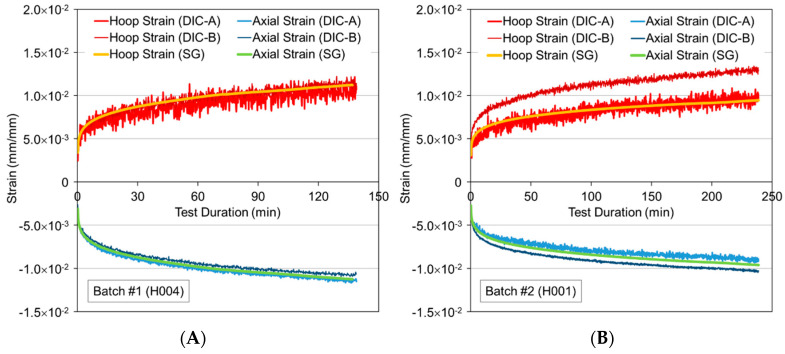
Comparison of DIC and strain gauge (SG) measurements for pure hoop tensile creep tests for representative samples from Batch #1 (**A**) and Batch #2 (**B**).

Figure 8 shows examples of strain versus time curves for measurements obtained from strain gauges and DIC. While DIC measurements exhibited significant noise, the qualitative agreement between the different measurement techniques provides confidence in the strain gauge readings. Data obtained from DIC-A and strain gauge measurements were found to be generally also in good agreement quantitatively, while in some tests, data from DIC-B deviated from the other readings. Differences in DIC-B data, as well as noise in DIC data, was attributed to optical distortions caused by the polycarbonate shield. Hence, strain gauge readings were deemed more reliable, and further analyses were based on corresponding data. It is worth pointing out that first stage and second stage creep can be observed from the graphs plotted in Figure 8; none of the specimens experienced tertiary creep, supposedly due to the low loading conditions.

### 3.2. Creep Strain Modelling

**Figure 9:**
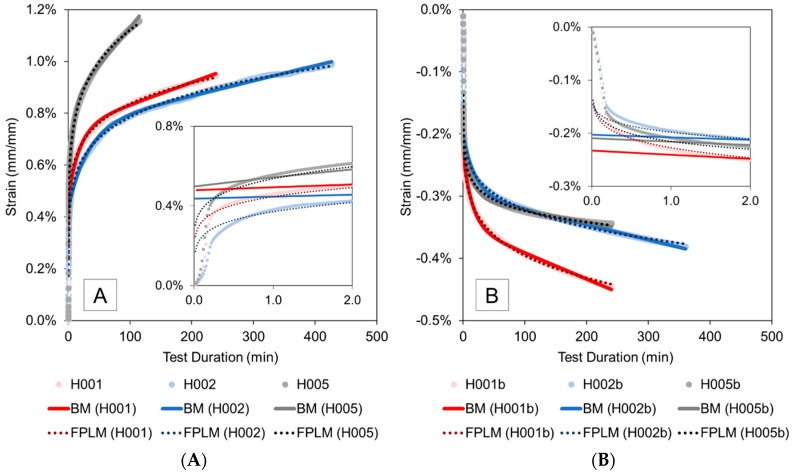
Comparison of the model data and experimental results for (**A**) pure hoop tensile and (**B**) pure axial compression tests (BM: Burgers model; FPLM: Findley’s power law model). Insets show the initial 2 min of testing.

Using the BM and FPLM, the data from pure hoop tensile and pure axial compression creep tests were studied further. The model parameters were obtained by curve fitting experimental data using MATLAB’s built-in “Curve Fitting” app (MathWorks, Natick, MA, USA). The error sum of squares and root mean square error values were close to zero while the adjusted *R*^2^ values were close to unity (ranging between 0.8773 and 0.9887, and 0.9873 and 0.9992 for the BM and FPLM, respectively), which confirms that the models are a good fit for the collected data. Figure 9 depicts the agreement between the models and the experimental results for the pure hoop tensile and pure axial compression tests. For clarity, only one specimen each from short-term, intermediate and longer-term creep tests is shown in the graphs. It should be noted that the strains computed by the BM and FPLM show comparatively high deviations from the experimental strain values during the initial 10-s ramp-up period. After the ramp-up period the BM initially produces higher deviations from experimental values than the FPLM (see insets in Figure 9); however, both models predict strains within 10% of experimental results as the tests progressed. Overall, the average of the absolute percent difference between the experimental results for both models from after ramp-up to the end of hold period with constant stress is under 1%. Both models demonstrate good agreement with experimental results for both tensile and compression loading. These findings were expected since both models have been shown to perform well for fitting creep data of fiber-reinforced HDPE composite materials [39,40,41]. On average, the FPLM provides data that are closer to the experimental results of this study as compared to the BM. Wang et al. [41] also observed FPLM to fit test data better than the BM in their comparison of the models for curve fitting creep data of a fiber-reinforced HDPE material.

### 3.3. Strain Rates

**Figure 10:**
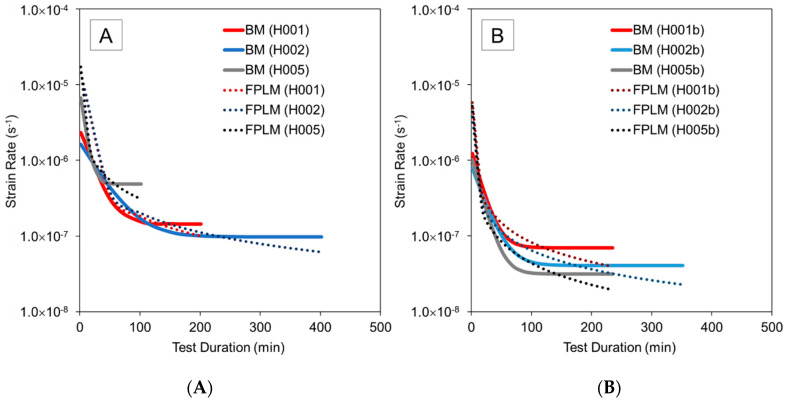
Strain rate computed using Burgers model (BM) and Findley’s power law model (FPLM) for (**A**) pure hoop tensile and (**B**) pure axial compression tests.

Calculating strain rates from experimental data is greatly affected by noise. Since the BM and the FPLM provided strain data that closely matches the experimental results, it is expedient to compute strain rates using the data obtained from these models. Figure 10 compares the strain rate data computed using the BM and FPLM for the creep tests under pure hoop tensile loading and pure axial compression. A phase of decreasing strain rate is initially observed which corresponds to the primary creep stage [42]. It is further shown that the pure hoop tensile tests, having a higher applied stress of 10 MPa, resulted in higher strain rates than the axial compression tests, which had a stress level of 5 MPa. The computed strain rates are lower than the steady-state strain rate for pure HDPE of 2.22 × 10^−6^ s^−1^ observed by Pereira et al. [43]. In their study the creep experiments were conducted at 3 MPa stress for only 10 min, nevertheless, the data provide indication that the GFR-HDPE material has a reduced strain rate compared to the pure polymer.

As shown in Figure 10, the BM computed strain rates eventually level off to a constant value regardless of test duration. However, the experimental data show that the strain rates for short-term tests are still decreasing steeply and do not reach steady-state creep. The FPLM computed strain rates continue to decrease gradually. It was therefore concluded that the FPLM better represents the behavior of the material, which is supported by the fact that the data from the FPLM had a lower percentage error from the experimental results compared to the BM. Consequently, remaining analyses in this study were conducted employing the FPLM.

### 3.4. Poisson’s Ratio

**Figure 11:**
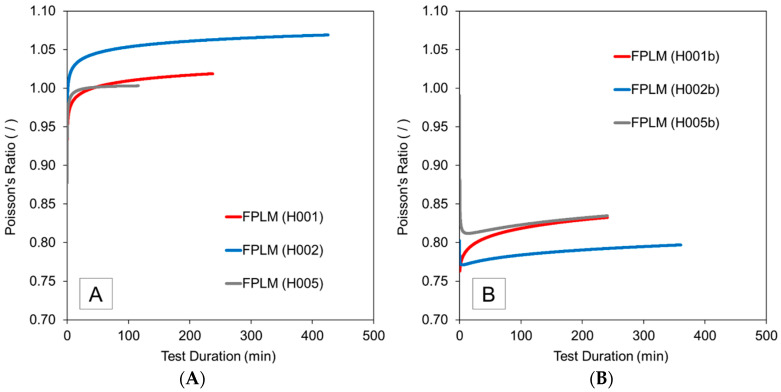
Poisson’s ratio computed using Findley’s power law model (FPLM) for (**A**) pure hoop tensile and (**B**) pure axial compression tests.

Figure 11 shows Poisson’s ratios that were computed using the FPLM for the pure hoop tensile creep tests and axial compression tests. The Poisson’s ratio for isotropic materials has an upper limit of 0.5. However, this restriction does not apply to anisotropic materials [44]. In comparison, the Poisson’s ratio calculated from classical lamination theory ranges from 0.83 to 0.88, corresponding to higher and lower Young’s moduli for both the glass fiber and HDPE, respectively (as listed in Table 3). All specimens exhibited an increase in Poisson ratio as the creep tests proceeded. The Poisson’s ratio eventually increases above unity, indicating that axial strain is higher than the hoop strain despite the loading conditions being predominantly in the hoop direction. This counterintuitive behavior has been observed before in ±45° pipes pressurized in pure hoop [45], as well as ±45° laminates loaded in pure tension [46]. In both of these studies, the highest strains before failure were in the direction perpendicular to the applied load which supports the results captured in the current study. It is presumed that changes in Poisson’s ratio are strongly influenced by fiber realignment effects in the specimens, which is consistent with classical lamination theory predictions that yield an increasing Poisson’s ratio as the angle between the fibers and the loading direction decreases from 45° for angle-ply laminates [44].

The Poisson’s ratio data for axial compression tests in Figure 11 also indicate an increasing trend with test duration, yet the Poisson’s ratio does not exceed unity, meaning the final axial strain is higher than the hoop strain for axial compression. Again, similar observations have been reported in the technical literature, i.e., results from experiments conducted by Elghazouli et al. [47] for a composite pipe with a ±45° fiber angle subjected to axial compression agree with the trend in final strains observed in the present study. Note that for some samples an initial drop in Poisson’s ratio was detected. The reason for this drop is not entirely clear but it is suspected that samples may not have seen full creep recovery during the rest period following the pure hoop tensile creep tests.

### 3.5. Creep Compliance

**Figure 12:**
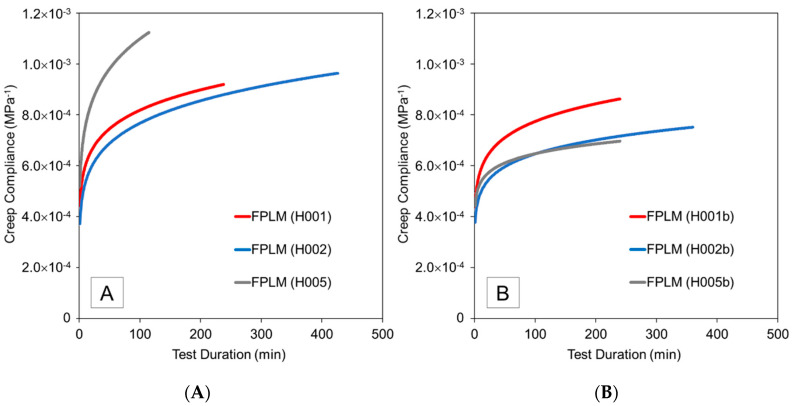
Creep compliance computed using Findley’s power law model (FPLM) for (**A**) pure hoop tensile and (**B**) pure axial compression tests.

The change in creep compliance as computed by the FPLM for pure hoop tensile creep tests and axial compression tests is depicted in Figure 12. The observed increase in creep compliance, which is the ratio of time-dependent strain to applied stress, was described also in other works on creep of fiber-reinforced polymer composites [26,48]. Indeed, such behavior is expected since stress is held constant while the material experiences creep strain over time. Elleuch and Taktak [49] observed a creep compliance above 2 × 10^−3^ MPa^−1^ for pure HDPE subjected to 10 MPa pure tensile stress; this value was observed at approximately 150 min into the creep test. The value for pure HDPE is at least two times larger than creep compliances determined from the present test data. The comparison between the present work and the study by Elleuch and Taktak supports previous research [27] which demonstrated that reinforcing a polymer with fibers can result in overall lower creep compliance.

In the case of pure axial compressive loadings, the GFR-HDPE also demonstrated a lower creep compliance compared to the pure HDPE used by Elleuch and Taktak [49]. For pure HDPE, Elleuch and Taktak observed qualitatively and quantitatively similar creep compliance curves for applied tensile and compressive stresses ranging up to 10 MPa, leading them postulate that the material behavior can be characterized by master curves for tensile and compressive loadings. In the present study, creep compliance was noticeably lower for axial compression tests than for the pure hoop tensile tests, and results exhibited poor quantitative similarity even for samples from the same batch. It is postulated that these differences in behavior of the GFR-HDPE arise from the presence of the fiber reinforcement, which seems to impart greater variability between samples and a dependency of creep compliance on the applied stress level and/or type of loading (tensile versus compressive), which may be associated with interactions between the polymer matrix and the fiber reinforcement phase, such as the process of fiber realignment and maybe even fiber-matrix debonding. Further research with a greater sample populations is needed to explore these postulates.

## 4. Conclusions

A commercial filament winding system was modified and employed to fabricate tubular coupons from glass fiber reinforced high density polyethylene (GFR-HDPE) tape. The quality of the tubes was verified through dimensional measurements, crush tests, and the analysis of their microstructure. Besides manufacturing the coupons, a creep testing setup was developed and utilized to explore the creep behavior of the material under different loading conditions.

Creep tests ranging from 2 to 7 h in length were conducted in a multi-axial testing apparatus. Specimens were tested under comparatively low loads at room temperature, i.e., pure hoop tensile tests at 10 MPa stress, followed by 5 MPa pure compressive tests after a resting period. Strain gauges and digital image correlation were employed to measure strain during the tests. The material exhibited noticeable creep behavior even at low loads. While specimens from different fabrication batches displayed different creep properties, specimens within the same batch had consistent test results. The differences between batches suggest that varying manufacturing parameters such as processing temperature affected the materials’ creep properties.

The experiments gave confidence in the accuracy of strain gauge measurements since these were in good agreement with predicted initial strain values obtained by classical lamination theory and the DIC measurements. However, overall DIC measurements experienced greater noise and occasionally deviated from anticipated values, which was attributed to optical distortions in the experimental setup (clear safety shield).

Overall, creep testing yielded reliable data which was used to determine parameters for prominent creep models, i.e., the Burgers model and the Findley’s power law model. The fit of experimental data to both models indicated that Findley’s power law model provided a closer prediction the obtained creep data than the Burgers model. The analyses of post-processed test data showed that GFR-HDPE specimens demonstrated improved creep resistance, evaluated by strain rate and creep compliance, compared to creep results of pure HDPE found in the technical literature. Observed changes in Poisson’s ratio during the tests suggest that material creep was accompanied by fiber realignment. The testing further revealed a dependency of the GFR-HDPE creep response on the applied load level and/or load type (tensile versus compressive), which was reported to be absent in pure HDPE polymer at comparatively low stress conditions.

In conclusion, the experiments and data analyses demonstrated that the developed creep testing setup is effective in providing repeatable test results for tubular coupons, avoiding edge effects that may mar findings obtained from flat coupons. The setup may thus serve as an expedient means for describing material behavior for input into design processes of GFR-HDPE structures that experience creep deformation during service, such as pressure piping. It is recommended to focus future studies on using creep test data as input for numerical modelling techniques, since such models may overcome limitations of analytical models, i.e., the inability to account for fiber realignment effects. Furthermore, it would be worthwhile to study the effect of different loading conditions, such as higher loads and longer test durations, on the creep response of GFR-HPDE materials.

## Figures and Tables

**Table 1:** Heating setting for tape winding two batches of thermoplastic fiber-reinforced polymer composites (TP-FRPC) tubular coupons.

Air Temperature * (Heater Setting)	Layers	
	Batch #1	Batch #2
400 °C (9)	1	1
400 °C (9)	2 to 7	2 to 4
420 °C (9.5)	8 to 10	5 to 8
440 °C (10)	-	9 to 10

* at a nozzle airflow of 410 L/min.

**Table 2:** Test matrix for creep experiments.

Test Name	Specimen Inner Diameter (mm)	Specimen Wall Thickness (mm)	Internal Pressure (MPa)	Axial Force (N)
H001	61.92	3.64	1.176	−3829
H001b			N/A	−3829
H002	69.14	3.65	1.178	−3548
H002b			N/A	−3757
H003	69.14	3.59	1.168	−3516
H004	69.20	3.65	1.181	−3553
H005	69.01	3.60	1.165	−3495
H005b			N/A	−3699

**Table 3:** Elastic properties of composite material constituents used for initial strain predictions.

Property	Glass Fiber	HDPE
Young’s modulus range (GPa)	68.9–85.0	0.7–1.34
Poisson’s ratio	0.20	0.46

## References

[1] Bogner B.E. (2008). Large diameter pipe: Lasting function in a world of growth. Reinf. Plast..

[2] Ossai C.I., Boswell B., Davies I.J. (2015). Pipeline failures in corrosive environments—A conceptual analysis of trends and effects. Eng. Fail. Anal..

[3] Koch G.H., Brongers M.P., Thompson N.G., Virmani Y.P., Payer J.H. (2002). Corrosion Cost and Preventive Strategies in the United States.

[4] Composite Pipe Applications and Design Factors to Extend System Life. www.lbcg.com/media/downloads/events/503/12-00-otto-comin-flexpipe-systems.8597.pdf.

[5] The Markets: Oil and Gas 2016. www.compositesworld.com/articles/the-markets-oil-and-gas-2016.

[6] Fellet M., Nyborg R. (2018). Understanding corrosion of flexible pipes at subsea oil and gas wells. MRS Bull..

[7] FlexFlow Linepipe Produced Water Transfer Line Installation. www.shawcor.com/about/success-stories/flexflow-linepipe-produced-water-transfer-line-in.

[8] LeBlanc J., Sternisha M. (2016). Fiberglass pipe is helping solve the world’s drinking water shortage. Pipelines.

[9] Rafferty K.D., Lienau P., Lunis B. (1991). Piping. Geothermal Direct-Use Engineering and Design Guidebook.

[10] Papanicolaou G.C., Zaoutsos S.P., Guedes R.M. (2011). Viscoelastic constitutive modeling of creep and stress relaxation in polymers and polymer matrix composites. Creep and Fatigue in Polymer Matrix Composites.

[11] Kaddour A.S., Hinton M.J., Soden P.D. (2003). Behaviour of ±45° glass/epoxy filament wound composite tubes under quasi-static equal biaxial tension-compression loading: Experimental results. Compos. Part B.

[12] Yu K., Morozov E.V., Ashraf M.A., Shankar K. (2017). A review of the design and analysis of reinforced thermoplastic pipes for offshore applications. J. Reinf. Plast. Compos..

[13] Brauner C., Herrmann A.S., Niemeier P.M., Schubert K. (2017). Analysis of the non-linear load and temperature-dependent creep behaviour of thermoplastic composite materials. J. Thermoplast. Compos. Mater..

[14] Ebewele R.O. (2000). Polymer Science and Technology.

[15] Lakes R. (2009). Viscoelastic composite materials. Viscoelastic Materials.

[16] Ashby M.F., Jones D.R.H. (2012). Engineering Materials 1—An Introduction to Properties, Applications, and Design.

[17] Yoon S.H., Oh J.O. (2015). Prediction of long term performance for GRP pipes under sustained internal pressure. Compos. Struct..

[18] Faria H., Guedes R.M. (2010). Long-term behaviour of GFRP pipes: Reducing the prediction test duration. Polym. Test..

[19] Tuttle M.E., Brinson H.F. (1986). Prediction of the long-term creep compliance of general composite laminates. Exp. Mech..

[20] Ghorbel I. (1996). Durability of closed-end pressurized GRP filament wound pipes under hygrothermal aging conditions. Part II: Creep tests. J. Compos. Mater..

[21] Fliegener S., Hohe J., Gumbsch P. (2016). The creep behavior of long fiber reinforced thermoplastics examined by microstructural simulations. Compos. Sci. Technol..

[22] Yang Z., Wang H., Ma X., Shang F., Ma Y., Shao Z., Hou D. (2018). Flexural creep tests and long-term mechanical behavior of fiber-reinforced polymeric composite tubes. Compos. Struct..

[23] Findley W.N., Lai J.S., Onaran K. (1976). Creep and Relaxation of Nonlinear Viscoelastic Materials—With an Introduction to Linear Viscoelasticity.

[24] Scott D.W., Lai J.S., Zureick A.-H. (1995). Creep behavior of fiber-reinforced polymeric composites: A review of the technical literature. J. Reinf. Plast. Compos..

[25] Schledjewski R. (2009). Thermoplastic tape placement process—In situ consolidation is reachable. Plast. Rubber Compos..

[26] Xiao X. (1989). Studies of the viscoelastic behaviour of a thermoplastic resin composite. Compos. Sci. Technol..

[27] Nguyen D.H., Ogale A.A. (1991). Compressive and flexural creep behavior of carbon fiber/PEEK composites. J. Thermoplastc Compos. Mater..

[28] Mittelstedt C., Becker W. (2007). Free-edge effects in composite laminates. Appl. Mech. Rev..

[29] International Organization for Standardization (2003). Plastics—Determination of Creep Behaviour—Part 2: Flexural Creep by Three-Point Bending.

[30] International Organization for Standardization (2015). Plastics Piping Systems—Glass-Reinforced Thermosetting Plastics (GRP) Pipes—Determination of Time to Failure under Sustained Internal Pressure.

[31] International Organization for Standardization (1997). Plastics Piping Systems—Glass-REINFORCED Thermosetting Plastics (GRP) Pipes—Determination of the Creep Factor under Dry Conditions.

[32] Funck R., Neitzel M. (1995). Improved thermoplastic tape winding using laser or direct-flame heating. Compos. Manuf..

[33] Clancy G., Peeters D., Oliveri V., Jones D., O’Higgins R.M., Weaver P.M. (2019). A study of the influence of processing parameters on steering of carbon fibre/PEEK tapes using laser-assisted tape placement. Composites Part B.

[34] Mazumdar S.K., Hoa S.V. (1996). Determination of manufacturing conditions for hot-gas-aided thermoplastic tape winding. J. Thermoplast. Compos. Mater..

[35] Behjat Y., Cheng J.J., Polak M.A., Asce M., Penlidis A. (2014). Effect of molecular structure on the short-term and long-term mechanical behavior of high-density polyethylene. J. Mater. Civ. Eng..

[36] Kaw A. (2006). Mechanics of Composite Materials.

[37] Agarwal V., Mccullough R.L., Schultz J.M. (1996). The thermoplastic laser-assisted consolidation process—Mechanical and microstructure characterization. J. Thermoplast. Compos. Mater..

[38] Xia X.C., Zhang Q.P., Wang L., Feng J.M., Fu X.R., Yang M.B. (2014). Role of gas cooling time on crystalline morphology and mechanical property of the HDPE parts prepared by gas-assisted injection molding. Colloid Polym. Sci..

[39] Xu Y., Wu Q., Lei Y., Yao F. (2010). Creep behavior of bagasse fiber reinforced polymer composites. Bioresour. Technol..

[40] Xu H.L., Cao Y., Wang W.H., Wang Q.W. (2016). Creep model of natural fiber reinforced polymer composite. Mater. Sci. Forum.

[41] Wang W.-H., Huang H.-B., Du H.-H., Wang H. (2015). Effects of fiber size on short-term creep behavior of wood fiber HDPE composites. Polym. Eng. Sci..

[42] Nomula S.S.R., Rathore D.K., Ray B.C., Prusty R.K. (2019). Creep performance of CNT reinforced glass fiber/epoxy composites: Roles of temperature and stress. J. Appl. Polym. Sci..

[43] Pereira A.A.C., D’Almeida J.R.M., Castro T.M.L. (2018). Evaluation of short-term creep behavior of PE-HD after aging in oil derivatives. Polym. Polym. Compos..

[44] Peel L.D., Abrams F., Patel J. P97 Investigation of high and negative Poisson’s ratio laminates. Proceedings of the 50th International SAMPE Symposium and Exhibition.

[45] Al-Salehi F.A.R., Al-Hassani S.T.S., Hinton M.J. (1989). An experimental investigation into the strength of angle ply GRP tubes under high rate of loading. J. Compos. Mater..

[46] Liang Y., Wang H., Gu X. (2013). In-plane shear response of unidirectional fiber reinforced and fabric reinforced carbon/epoxy composites. Polym. Test..

[47] Elghazouli A.Y., Chryssanthopoulos M.K., Spagnoli A. (1999). Experimental response of glass-reinforced plastic cylinders under axial compression. Mar. Struct..

[48] Yao J., Ziegmann G. (2006). Equivalence of moisture and temperature in accelerated test method and its application in prediction of long-term properties of glass-fiber reinforced epoxy pipe specimen. Polym. Test..

[49] Elleuch R., Taktak W. (2006). Viscoelastic behavior of HDPE polymer using tensile and compressive loading. J. Mater. Eng. Perform..

